# Employment Growth through Labor Flow Networks

**DOI:** 10.1371/journal.pone.0060808

**Published:** 2013-05-02

**Authors:** Omar A. Guerrero, Robert L. Axtell

**Affiliations:** 1 Department of Computational Social Science and Center for Social Complexity, George Mason University, Fairfax, Virginia, United States of America; 2 Santa Fe Institute, Santa Fe, New Mexico, United States of America; Cinvestav-Merida, Mexico

## Abstract

It is conventional in labor economics to treat all workers who are seeking new jobs as belonging to a labor pool, and all firms that have job vacancies as an employer pool, and then match workers to jobs. Here we develop a new approach to study labor and firm dynamics. By combining the emerging science of networks with newly available employment micro-data, comprehensive at the level of whole countries, we are able to broadly characterize the process through which workers move between firms. Specifically, for each firm in an economy as a node in a graph, we draw edges between firms if a worker has migrated between them, possibly with a spell of unemployment in between. An economy's overall graph of firm-worker interactions is an object we call the *labor flow network* (LFN). This is the first study that characterizes a LFN for an entire economy. We explore the properties of this network, including its topology, its community structure, and its relationship to economic variables. It is shown that LFNs can be useful in identifying firms with high growth potential. We relate LFNs to other notions of high performance firms. Specifically, it is shown that fewer than 10% of firms account for nearly 90% of all employment growth. We conclude with a model in which empirically-salient LFNs emerge from the interaction of heterogeneous adaptive agents in a decentralized labor market.

## Introduction

Employment dynamics are the product of complex interactions taking place inside and between firms. In labor markets, human resources are continuously reallocated across firms, industries, and regions. In labor economics it is conventional to aggregate job hirings and job separations (both voluntary and involuntary) across companies to get pools of job changers and the unemployed [1]. The sizes of these pools are then conceived of as being determined by rate processes over these pools [2]. In reality, hiring and separation occur at individual companies and important information about the varieties of firm behavior is lost in the process of aggregating labor data into pools, with otherwise comparable firms experiencing quite different labor turnover. For instance, understanding how micro-dynamics affect aggregate variables (such as employment growth) from a disaggregate perspective is an ongoing challenge. We demonstrate that the science of complex networks can be helpful in tackling this problem.

Over the past fifteen years the important role of networks in human society has become readily apparent, from the topology of the internet [3], [4] to the rise of social media. In many areas of science the growing availability of micro-data has made possible the systematic study of networks (e.g., citation networks [5]) while in other domains the growth of computing power has led naturally to network conceptions of social processes (e.g., epidemiology [6], [7]). In economics the study of networks has essentially revolved around strategic concerns and a game theoretic orientation has become the norm [8]–[10]. Fewer studies analyze economic networks that are the side product of other kind of interactions, instead of being the intended consequence of strategic behavior. Networks of companies are an example of such structures. The first studies of large-scale complex networks of firms were made for the Japanese economy, including ownership networks [11] and costumer-supply networks [12], [13]. More recent studies have done similar analyses for the US [14].

Here we blend these motivations for studying networks, using newly available micro-data and the ability to work with large-scale, complex networks computationally, to study labor dynamics. Here we characterize a LFN for an entire economy. We also provide a model that generates many of the properties of the empirical LFN from economic behavior. The data do not tell us about the motivations of individual workers for changing jobs. However, we are able to develop a model that is consistent with the data in which workers act in their own self-interest strategically in seeking better employment opportunities.

## Materials and Methods

In this section we describe the datasets, define the concept of a labor flow network, describe the tool of ‘null models’, introduce measures of employment growth, and present a model.

### Primary Dataset

We used a comprehensive dataset about labor and companies in Finland. Most of our results derive from it. It contains the universe of employed individuals in Finland and their employers (both from the private and public sectors). FLEED's employer units are enterprises, defined as *economic activity carried on by one or more persons for profit-making purposes*. Although this is a broader definition than the one conventionally used for firms, we will use it interchangeably since it does not change our ability to measure employment growth. Unless otherwise specified, all the analysis was conducted using this dataset.

This dataset is called Finnish Longitudinal Employer-Employee Dynamics (FLEED) and is provided by Statistics Finland. FLEED contains annual registries of every permanent resident in Finland that is employed. These records are constructed from administrative registries by extracting the social security number of the employed individuals and the identification number of their respective employers. Individuals and firms are anonymized through unique identifiers. FLEED consists of annual panels with pairs of identifiers: employee and employer. Each panel is constructed using the record available for each employed individual on the last day of the year. Therefore, if an individual is unemployed during the last day of the year, she will not appear in corresponding panel. FLEED only captures annual movements of individuals and does not distinguish between workers who underwent unemployment spells and those who were job-changers. For most of the analysis, we used FLEED's panels from 2005, 2006, 2007, and 2008. On average, each panel contains 230,000 employer identifiers.

We merged FLEED with Statistics Finland's Business Registries in order to obtain accurate information about the size and age of each employer. These registries consist of annual panels of the universe of firms in Finland. They provide information about number of employees, year of birth, and year of death. In order to prevent identification of individual firms through their size, this variable was treated with a log-normally distributed random noise by Statistics Finland. We linked these records to FLEED's employers' identifiers.

### Support Dataset

We used a sample dataset from Mexico that we obtained in order to evaluate the robustness of our results. Although it is similar in size to the primary set, it does not comprise the universe of Mexican firms and workers. Its nature and sampling method makes it prone to be biased in ways that the primary dataset is not. Nevertheless, it is a useful source of information to evaluate the robustness of our results. It was used exclusively for the Robustness section in this paper.

We obtained this micro-dataset from the Mexican Institute for Social Security (IMSS, after its Spanish acronym). Like in the Finnish registries, the IMSS data contains records with anonymized pairs of individuals and employers. This set only contains records from the formal private sector. Approximately half of the employees in the private sector are not registered with the IMSS. Thus, they are considered informal workers. Additionally, nearly 16% of all Mexican workers are state-employed, so they are not in the IMSS records either.

The IMSS dataset has daily resolution. When an individual joins the formal private sector, a record is written in order to link her to the current employer and the exact date she joined is recorded. If a worker joins a different employer, the new pair of identifiers is recorded with the date of the movement. The sample consists of 1% of all registered individuals in 2008. Once individuals have been sampled, their entire labor history was extracted from the database, i.e. for each individual, all the identifiers of her past employers are listed with the respective days in which she joined them. Therefore, an employer appears in the dataset as long it employs at least one individual from the sample. In total, our sample consists of 400,000 individuals, with an average of 10 records each. Roughly speaking, this dataset contains 270,000 employer identifiers.

### Labor Flow Networks

Consider a network in which the firms are vertices and an edge is drawn between firms whenever a person has worked at one company and subsequently moved to another. For an economy as a whole we call this the *labor flow network* (LFN). We use FLEED data to construct the LFN of Finland. The motives and means of individuals to move from one employer to another are diverse, ranging from economic incentives and unemployment spells to personal contacts and geographic relocation. This network implicitly captures most of these factors since it is constructed from the actual labor flows of the economy. We believe that studying its structure and its relation to other elements of firms' dynamics can improve our understanding of labor dynamics and the role of firms in employment growth.

The construction of a LFN is rather simple. For a selected period, we count the total flows of labor between every two firms in both directions. Although this is a directed network, we found that the most interesting insights come from studying its structural properties as an undirected graph. Therefore, our analysis uses algorithms for undirected networks (with exception of in-degree and out-degree centralities).

### Null Models

When data is available for a particular network, it is the case that such a network is one realization of a social process. In our case, our main LFN is the reallocation of labor across Finnish firms between the last day of 2005 and 2008. If we were able to let the Finnish workers and companies to search again, assuming the same conditions of 2005–2008, it is possible that some properties of our LFN would not be found in the new LFN. In that case these properties are not robust and we cannot draw correct inferences from them. However, labor and firm dynamics at that scale are not easy to replicate under an experimental setting. For these situations, null models are useful to draw better inferences.

Null models were introduced by [15] and, examples of how they are used with economic data can be found in [16], [17]. The main idea is to take the network provided by the data and randomize its structure while fixing some of the properties of the nodes (usually the degree). We need to create a sample of these randomized networks in order to estimate the parameters of interest. In our case, we generated 50 randomized LFNs. We indicate when an estimate was drawn from this procedure.

### Employment Growth and Firms

In order to analyze employment growth we employ metrics from the small-business literature. Such measures typically extend for a defined period (3 or 4 years), and are based on changes in the sizes of companies. In this study, firms' sizes are measured as the number of employees. Since we are analyzing data from a European country, we use the metric developed in the OECD/Eurostat methodology [18]. Call *S_t_* the size of a firm at time *t*, and *S_0_* its initial size. Then *ΔS* is the *average annual growth* of such a firm for the period between *t* = 0 and *t = T*:
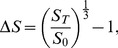
(1)Growth is not defined for firms with zero employees initially, as is evident from (1). We added one employee to all firms in order to compute (1) for firms with zero employees. Another common problem is defining growth for companies that ceased to exist at *t<T*, and for those that were created at *t>0*. We resolved this issue by focusing our analysis on those firms that were born in 2005 or before and still existed in 2008. We call them *survivors*.

Following (1), we measured growth of all *survivors* and classified them, according to three standard taxonomies employed in small-business research and added two more.

**Positive growth firm (PGF):** *A survivor firm with positive growth.*

**Labor flow network firm (LFNF)**: *A PGF with at least one connection to another firm in the LFN*.

**High-growth firm (HGF)** [18]: *A PGF with average annualized growth greater than 20% per year*.

**Gazelle firm (GF)** [18]: *A HGF that is at most 5 years old*.

**High-impact firm (HIF)** [19]: *A HGF with an employment growth quantifier (EGQ) of two or more*.

The EGQ is defined by [19] as: *the product of the absolute and percent change in employment, expressed as a decimal*. The definition of a HIF has been slightly modified (by adding the age requirement) to use the OECD definition of a GF.

### Agent-based Model

As part of our investigation to model the LFN formation process, we used an agent-based economic model that has proven to be robust for reproducing multiple patterns in firm micro-data [20], [21]. The model consists of a heterogeneous population of agents interacting through a team (or firm) formation game. Firm output determines the income of each agent. Agents have Cobb-Douglas preferences for income and leisure. Two key elements of the model are that (*i*) firm production functions have increasing returns to total efforts, and (*ii*) for large enough firms, variability of individual efforts leads to instability. Large firms are not stable because each agent's compensation is imperfectly related to its effort level, making free-riding possible. Highly productive agents eventually leave large firms and such firms eventually decline; for details see [20], [21].

This model generates job-to-job flow dynamics, involving agents switching between firms. We ran the model and wrote employer-employee matched records for each agent, generating artificial micro-datasets that were analyzed in the same way as the empirical data.

Roughly speaking, Finland has a work force of 2.5 million individuals. This is a manageable size for a high-resolution agent-based model. We calibrated the model to have a one-to-one scale with the Finnish labor force. We adjusted parameters of mobility to match the density of the labor flows for a period of three years post-transient. We ran 50 instantiations of the model and collected the respective micro-data. We submitted the artificial micro-data to the same analysis as the empirical one and show the results in the Emergence of Labor Flow Networks section in this paper.

## Results

In this section we present the results obtained for the Finnish labor flow network (LFN). First, we characterize its topology. Next, we show evidence of international robustness of these findings. Then, we present new evidence of correlations between the economic characteristics of firms and their structural position in the LFN. After, we demonstrate that the LFN yields new structural information about the configuration of communities of firms in the economy. Last, we show that the LFN is useful to identify signs of potential employment growth. Finally, we present the results of a computational model at a one-to-one scale with the Finnish labor market.

### Labor Flow Network

**Figure 1:**
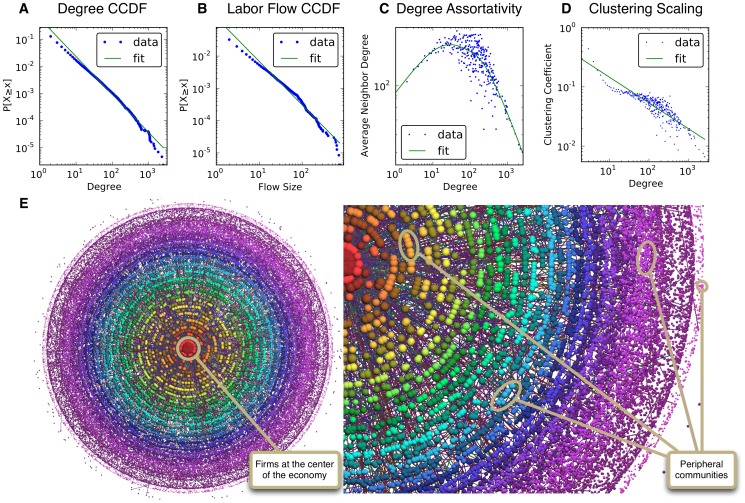
Topology of the labor flow network from Finland. Data from panels A and B were fitted using maximum likelihood estimation. Due to the unusual magnitude of the scaling parameter estimated for panel B, we do not think it is a power law. However, other skewed distributions do not produce better fits under the Kolmogorov-Smirnov criteria. We used kernel regression to identify critical regions in panel C. Estimations in panel D were made with OLS. Panel E shows the universe of firms in Finland. Only 1% of the edges are drawn. The size of the node represents the degree. The color identifies firms with the same *k*-core index. The image was produced with the visualization tool LaNet-vi and it shows the organization of the LFN into a core-periphery structure. Groups of firms are less tightly connected as we move from the center to the outside rings.

The Finnish LFN is a complex network. Figure 1 A–D shows statistical evidence that the topology of the network is not the product of purely random processes (as in an Erdös-Rényi type of network). Many of these patterns are described by a power-law relationship of the form
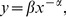
(2)where *α* is the scaling parameter and *β* a normalizing constant. If we are talking about a CDF, then *y* = Pr[*X*≥*x*] and *β = (x_0_)^α^*, where *x_0_* is the smallest unit. Otherwise, *x* and *y* are variables that are related in this way.

Labor flows are heavy-tailed. This means that extreme events involving large flows occur more often than would be expected if labor reallocations were normally distributed. The degree distribution follows (2) with *α = *3.19±0.003 (panel A in Figure 1). The size of the labor flows between pairs of firms (the LFN links) can be fitted to the same distribution with *α = *11.58±0.02 (panel B in Figure 1). An average Finnish firm in the LFN receives 2.95±0.07 workers from 3.08±0.03 firms and sends 2.95±0.08 to 3.12±0.04 firms.

As in other complex networks, the topology of the LFN encapsulates structural information about the labor market. This relates to the connectedness that nodes have in terms of their neighborhood. In labor dynamics, we interpret connectedness as the accessibility that an individual has to other firms, given the position of her last employer in the LFN. Accessibly to firms can have different connotations, e.g. geographical, social, educational, industrial, etc. What is important is the close relationship between access to firms and access to vacancies or job opportunities. We believe that this is the essence of the underlying mechanism that drives labor reallocation dynamics and employment growth.

We analyzed the tendency of firms to be connected to firms with similar number of connections by looking at the average neighborhood degree. If the two variables are positively (negatively) correlated the network is said to be assortative (disassortative). Panel C in Figure 1 shows that when Finnish firms have 35 connections of more, the LFN becomes disassortative (Pearson r of −0.22 sampling null models). This is a peculiar property since evidence from several datasets shows that *social* networks tend to have an assortative character while *technological* networks are often disassortative [22].

A firm is structurally important in its neighborhood if it provides workers with means of mobility. Such means take the form of human capital, social capital, geographical proximity, or any other asset that is valued enough to reallocate the outgoing labor. A firm that facilitates such mobility is key in labor reallocation because it becomes a middleman for its neighbors. Its absence restricts mobility to fewer firms, organized in smaller neighborhoods. We measured the clustering coefficients to estimate the structural importance of firms. Panel D in Figure 1 shows that clustering coefficients decrease with degree through a power law relationship. The estimated scaling parameter for the sampled null models is *α* = 0.64±0.003 with an R^2^ = 0.6; it is evidence of a *hierarchical structure* in a complex network [23]. Individuals in a hierarchical LFN have access to more firms, and more communities of firms, if their employers are in a higher level of the hierarchy. Here, clusters of nodes of a given level tend to be connected to a common cluster at a higher level. A cluster at an upper level becomes the broker of different communities from lower levels. This gives rise to a core-periphery structure of the economy.

We illustrate the core-periphery structure of the universe of Finnish firms in panel E of Figure 1 through a *k*-core decomposition. The visual representation [24] shows the organization of the LFN into different communities. The firms in the center are the ones at the top of the hierarchy. The further a firm is from the center, the lower its degree, its hierarchical level, and the tightness of its community. Job-search-wise, individuals in peripheral firms would have a harder time finding vacancies due to poor connectivity. Therefore, we are interested in investigating if there is any relationship between LFNs and employment.

### Robustness

Our results from the Finnish LFN represent the universe of firms and employed individuals in this country. A natural question is how robust are our findings regarding other countries? The purpose of this exercise is to show evidence that the topological characteristics of LFNs are robust across economies. Lack of more comprehensive data for Mexico prevents us from doing any kind of comparative analysis. However, in order to provide some background regarding structural differences between Finland and Mexico, Table 1 provides information about both countries during the period under study.

**Table 1:** Comparison between Finland and Mexico.

	2005		2006		2007		2008	
	Finland	Mexico	Finland	Mexico	Finland	Mexico	Finland	Mexico
**Population** 1	5,246.10	103,946.90	5,266.27	104,874.30	5,288.72	105,790.70	5,313.40	106,682.50
**GDP** 2	161.10	1,293.79	174.53	1,439.30	191.28	1,530.84	202.34	1,627.07
**GDP per capita** 3	30,707.92	12,460.54	33,140.17	13,740.55	36,167.38	14,485.97	38,080.46	15,267.18
**Employment rate** 4	68.52	59.65	69.58	60.95	70.46	61.06	71.25	61.31
**Self-employment rate** 5	12.67	35.54	12.90	34.46	12.65	34.34	12.85	33.94
**Part-time employment** 5	11.20	16.82	11.41	16.96	11.71	17.57	11.50	17.58
**Unemployment rate** 6	8.30	3.60	7.70	3.60	6.90	3.70	6.40	4.00
**Long-term unemployment** 7	24.88	2.33	24.82	2.55	22.97	2.72	18.17	1.65
**GDP per hour worked** 8	17.90	5.46	19.39	6.02	21.20	6.40	22.56	6.75
**GDP on R&D** 9	3.48	0.41	3.48	0.39	3.47	0.37	3.72	n/a
**Share of ICT in value added** 10	n/a	n/a	n/a	n/a	n/a	n/a	13.88	4.99
**Firms in MFG, less than 10** 11	83.73	11.49	83.28	11.00	83.15	9.45	81.64	n/a
**Firms in MFG, 50 to 249** 11	3.65	42.55	3.75	42.40	3.75	42.74	3.90	n/a
**Firms MFG, more than 250** 11	1.01	27.67	1.03	28.83	0.99	30.01	1.06	n/a
**Total number of firms**	246,149	3,001,610	291,560	n/a	322,108	n/a	332,586	4,724,892

Source: OECD.

1 Thousands.

2 Billion US dollars, current prices and PPPs.

3 US dollars, current prices and PPPs.

4 Share of persons of working age in employment.

5 As a percentage of total employment.

6 As a percentage of labor force.

7 Persons unemployed for 12 months or more as a percentage of total unemployed.

8 GDP per capita divided by the average of total hours worked annually by a person.

9 Percentage of GDP invested in research and development.

10 Percentage of the value added from the business sector that comes from the Information and Communication Technologies (ICT).

11 Number of firms in the manufacturing sector (MFG) with size in number of employees.

Although Mexico is considerably larger than Finland (both in population and GDP), the Nordic economy produces nearly twice as much as Mexico in per capita terms. The Finnish government provides unemployment benefits while in Mexico this is a private service that only a fraction of the formal sector acquires. Therefore, a Finnish worker has incentives to remain unemployed longer. This is reflected in some of Finland's indicators, such as the persistent higher unemployment rates, lower self-employment rates, and considerably higher long-term unemployment rates. One of the most noticeable structural differences is the role of technology and R&D. The share of ICT to value added from the Finnish business sector is higher than that of Mexico. Additionally, Finland is known to be one of the countries with the highest investment in R&D as a percentage of its GDP (more than 3%). Finally, the structure of the manufacturing sector in terms of the firm size distribution seems to be the opposite between the countries. Given that both economies show remarkable differences, we expect that evidence of common features between both LFNs would be an indicator of robustness.

We constructed a LFN using the support dataset from Mexico. Similarly to the Finnish data, we counted annual flows of labor at the end of each year of the sample period. This gave us a network with more than 160,000 Mexican firms, with similar density to the Finnish one.

**Figure 2:**
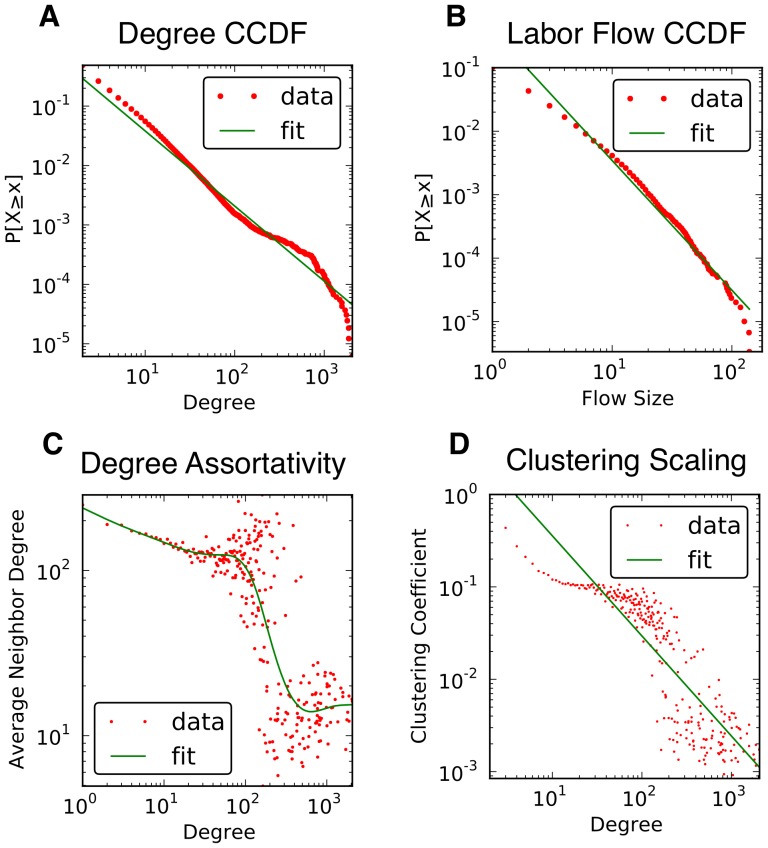
Topology of the labor flow network from Mexico. Data from panels A and B were fitted using maximum likelihood estimation. We used kernel regression to identify critical regions in panel C. Estimations in panel D were made with OLS. Each panel corresponds to one with the same letter in Figure 1.

Despite the differences between Finland and Mexico, and between the two datasets, it is remarkable that most of the statistical patterns describing the topology of the LFNs are robust across countries. Panel A in Figure 2 shows that the degree distribution of the Mexican LFN is (2), with *α = *3.17±0.005. The scaling parameter for the fitted labor flow size distribution (panel B of Figure 2) is quite high for a heavy tail: *α = *9.44±0.015. Panel C shows that this network is degree-disassortative, which is different from the Finnish LFN for firms with less than 35 connections. Finally, panel D provides evidence of a hierarchical structure in the Mexican network.

In a contemporary but independent study, Gianelle [25] constructed a LFN for the industrial region of Veneto, Italy. It consisted of all the workers and employers from the private sector of Veneto in the decade of the 1990's. His network had approximately 380,000 vertices. His results confirm the robustness of our findings regarding the Pareto distribution of the degree distribution of LFNs. A different network notion —bi-partite graphs of workers and firms— has been used for a similar analysis, finding Pareto degree distributions [26].

A different but related type of firms network is the supplier-customer ones. They have been studied comprehensively for Japan by [12], [13], where it was found that a network of 800,000 Japanese firms connected through economic transactions has the scale-free and hierarchical properties that we have found in our LFNs. Although these networks are of a different nature, they share common features with LFNs, suggesting the important role of firms' dynamics in economic systems.

### Networks and the Economy

An advantage of the FLEED dataset is that it can be merged with the Finnish business registries. This allowed us to go beyond simply characterizing the LFN topology in order to explore its relationship to the economic attributes of firms. We measured network properties for the 2005–2008 period in order to study their relationship to initial firm size and age. This helps to identify groups of firms that have particular structural roles in the LFN, firms that could be important for labor reallocation. All the results presented throughout the rest of our study are only for Finland.

**Figure 3:**
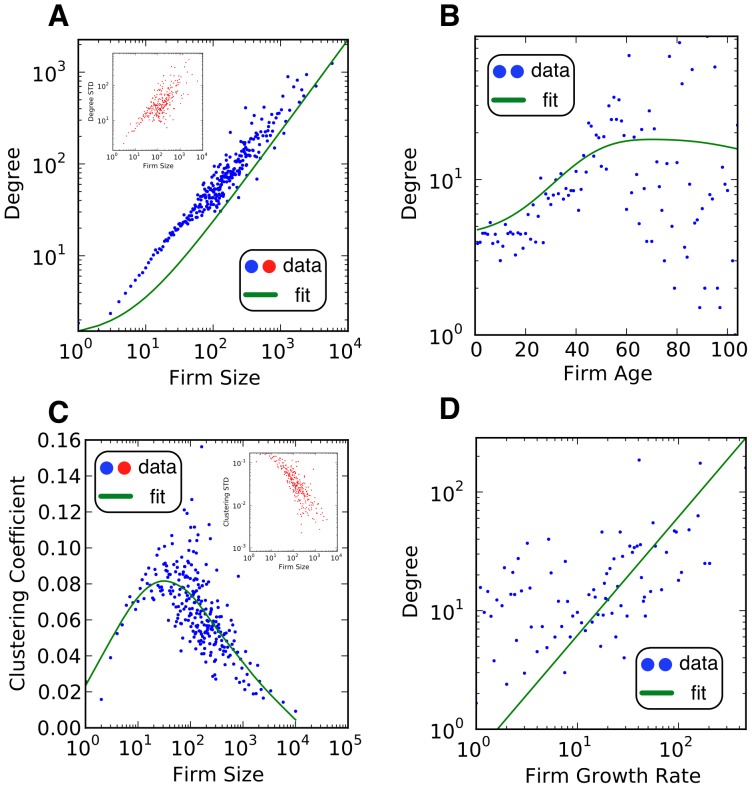
Correlations between network properties and economic variables. Critical regions in panels B and C were identified using kernel regressions.

We found that firm size and its degree are strongly correlated (Pearson r of 0.83), and that degree volatility increases as firms become larger (see panel A in Figure 3). However, degree and firm age are more complicated. Panel B in Figure 3 shows that the correlation between age and the average degree of the group exists only for firms less than 50 years of age (Pearson r of 0.79). This suggests that LFN formation is not determined by a pure Yule multiplicative process, a model commonly used to explain firms' growth [27]–[29] and more recently applied to scale-free networks [30]. It means that if the workers would tend to flow towards older firms, the latter would be hubs. This is clearly not the case for the Finnish LFN.

Firm size can tell us something about the structural importance of a firm in its neighborhood. Panel C in Figure 3 shows a positive correlation between size and average clustering coefficient for firms in groups with less than 28 employees (Pearson r of 0.67 after null model estimation). This relationship is negative for bigger firms (Pearson r of −0.72). Both small and large firms are middlemen in their communities. Large firms connect different groups of firms due to their higher position in the hierarchy. Small firms connect other small and medium-sized individual firms that, otherwise, would not be part of the LFN. Finally, panel D in Figure 3 suggests a positive relationship between degree and growth (Pearson r of 0.44).

### Sectors, Regions, and Communities

Identifying the target population of an employment policy is crucial for its success. Conventionally, firms are classified into industries or geographical regions at some particular level of aggregation. It is natural to think that firms in the same class tend to interact more with each other. Labor-wise, we would expect that a worker employed by Chrysler in Detroit would more likely get a job in Michigan and/or in a firm that falls into a related industrial classification. This does not seem to be the general case in today's labor market. Skill-based technical change of industrialized economies has increased the value of transferable and non-cognitive skills [31]. Today, it is rare to make a career in a single firm. Therefore, job changes are becoming more common. In the U.S., job-to-job flows have increased nearly 60% during the last decades [32]. The magnitude of job-to-job flows is nearly twice the number of employment-unemployment transitions [33], [34] for this country. In the case of Finland, industries and regions are more connected than ever [35], [36]. Thus, defining communities of firms to be the target of employment policies becomes a major challenge.

Labor policies that use standard classifications can incur in two types of errors when defining their target population: (*i*) exclusion of important firms and (*ii*) inclusion of irrelevant firms. Imagine that the wood manufacturing industry requires specific skills to use a new technology, and that firms are not able to provide enough training to meet their demand for trained labor. *On-the-job training programs* are a common solution. Here, the government finances wood manufacturers to provide training that meets their needs. However, now imagine the LFN tells us that this sector receives a substantial amount of labor from the recycling industry. Government financing training programs for firms from the recycling sector could improve the program's impact. Similarly, such a program could be more efficient by discarding wood manufacturers that do not show evidence of facilitating labor mobility. Hiring employees from neighboring firms (or sectors) can meet their demand for skills.

**Figure 4:**
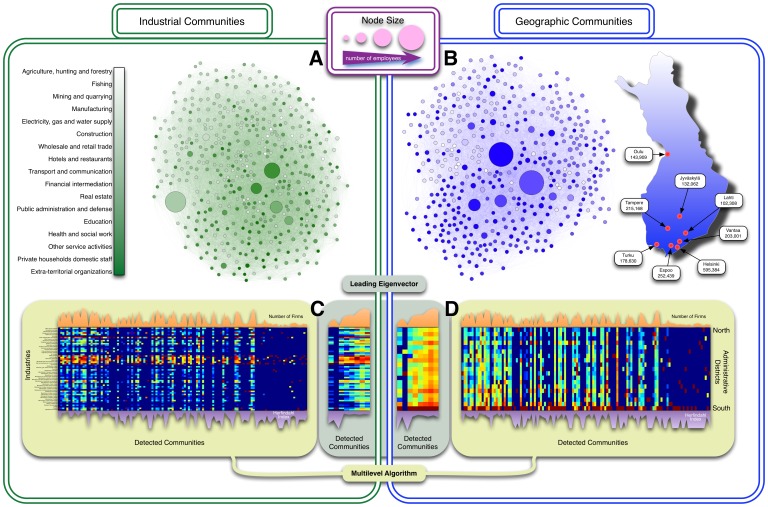
Communities of firms. Panels A and B provide a visual example of clusters in a reduced version of the LFN. The nodes represent industrial/geographical sectors as defined by the three-digit classifications from Statistics Finland. In panel B we provide information about the population of the eight largest cities in the country in order to illustrate the high concentration in southern districts. For both panel A and B, the color gradient corresponds to two-digit classifications. Their networks are laid out by the Force Atlas algorithm, which groups nodes according the strength of their ties. Panels C and D show the density matrices of the detected communities and the predefined industrial/geographical sectors. Each column has been normalized to illustrate the diversity of sectors in each community as a heat map. Cells represent the share of firms that each industry/region has in its respective community. The normalized total number of firms in each community is plotted on top of the heat maps. An inverted series of the Herfindahl–Hirschman index is plotted in charts bellow the heat maps.

Figure 4 presents the composition of the Finnish LFN by communities. Panel A and B illustrate how the conventional industrial classification do not match the arrangement generated by labor flows. The nodes in this chart represent entire industries at the three-digit classification level (larger nodes have more firms). The color gradient represents different industries at the two-digit classification level. If subsectors of a two-digit sector would exchange labor between themselves with higher propensity than with other sectors, then by grouping the nodes according to the “attraction” [37] represented by their edges should produce a layout where sectors of the same color are visually clustered. This is clearly *not* the case since the color gradient seems well mixed throughout the entire graph. Similar results occur for municipal classifications. In panel B we provide two and three-digit municipal aggregations, with the gradient representing the geographical position form north to south. In both industrial and geographical cases, non-labor based classifications do not appear to be representative of the community structure that underlies labor dynamics. We provide more rigorous evidence of this claim by analyzing the structure of the LFN at the firm level employing community detection algorithms.

Network community detection methods can complement other ways of characterizing the target population. There are many methods to detect such communites in networks [38]. A popular approach is the maximization of a modularity score, defined as *the difference between the number of edges inside a community and the expected quantity if such edges were placed at random* [39]. There are many algorithms that try to discover communities by partitioning the network and evaluating the modularity score. If the score is maximized and the partitions are not trivial, then we have evidence of communities.

In panels C and D of Figure 4 we present the results of two community detection algorithms that perform especially well in large-scale complex networks [40], [41]. The approaches use different ways of maximizing modularity. Therefore, their results differ. Our purpose here is to show the disparity between the communities that are identified using the LFN and the ones that are defined by industrial and geographical classifications. The discussion of which algorithm is more suitable for labor policies is something that we will leave for future inquiries. Vertical patters indicate the presence of communities composed of firms from different sectors or regions (type *i* error). Horizontal patterns indicate that there are different communities inside the same sector or region (type *ii* error). If there were a strong correspondence between detected communities and conventional categories, each community would show a high concentration index. We found that more than 70% of the communities detected via leading eigenvectors had a Herfindahl–Hirschman index (HHI) lower than 0.5, considered low. For communities detected using the multilevel algorithm, 79% had a HHI<0.6 for industries and 62% for geographical regions. We should point out that we used two-digit industrial and geographical classifications, which are the most aggregate. When using less aggregate classifications, the HHI drops because members of a community that used to belong to the same industry or region now belong to different ones.

### Employment Growth

A common policy approach to promote employment growth is the creation of programs that are targeted to firms that show signs of potential growth. Identifying these firms is often a challenge since understanding the underlying causes of a company's growth remains difficult.

Conventionally, the firm-dynamics literature employs the size and age of firms to identify their potential growth. For example, small-business advocates tend to argue that the size of a firm is negatively correlated with its growth. Since the work of David Birch [42]–[44], this has been the dominant paradigm in the small businesses literature. Birch argued that small rapidly growing businesses, which he called gazelle firms (GFs), were responsible for most employment creation. Acs [19] found that when we account not only for proportional grow, but also for the effect on employment, there is a subclass of gazelles that growth significantly more intensively; he calls them high-impact firms (HIFs). A posterior study [45] questioned the rationale behind such classifications by showing that the relationship between firm size and growth rate becomes irregular when controlling for age.

We propose the use of LFNs to identify potential growth of firms. Since LFNs capture structural information about the dynamics of labor, measures of the structural importance of a firm in the network may contain useful information. We ran a logistic regression of the probability that a firm experiences positive growth between 2005 and 2008, as a function of conventional predictors (initial size and age) and its LFN characteristics. The network covariates included measures of network centrality, clustering, and geographical assortativeness. In order to prevent spurious relationships, we computed the LFN metrics for the period 2002–2005.

Table 2 shows the network properties of firms can be used to identify the likelihood that a firm will experience positive growth. Once we introduce the network covariates, the marginal effect of the initial size becomes significant and consistent with the literature: larger firms are less likely to experience positive growth. Firms with higher in-degree and higher closeness are more likely to grow. Companies that are part of numerous shortest paths between two other firms are *less likely* to increase employment.

**Table 2:** LFN and employment growth.

	(1)	(2)	(3)	(4)	(5)	(6)	(7)
	Pr[Δsize>0]	Pr[Δsize>0]	Pr[Δsize>0]	Pr[Δsize>0]	Pr[Δsize>0]	Pr[Δsize>0]	Pr[Δsize>0]
**Size in 2005**	1.92e-05	2.07e-06	-0.000413***	-0.000333***	-0.000392***	-0.000384***	-0.000374***
	(2.33e-05)	(2.41e-05)	(1.00e-04)	(9.25e-05)	(9.96e-05)	(9.87e-05)	(9.83e-05)
**Age in 2005**		0.00140***	0.00139***	0.00117***	0.00115***	0.00114***	0.00110***
		(0.000118)	(0.000118)	(0.000119)	(0.000119)	(0.000119)	(0.000119)
**In-degree**			0.00287***	0.00152***	0.0101***	0.00983***	0.00924***
			(0.000454)	(0.000387)	(0.000804)	(0.000799)	(0.000798)
**Closeness**				0.00788***	0.00698***	0.00666***	0.00591***
				(0.000359)	(0.000363)	(0.000366)	(0.000379)
**Betweenness**					−3.499***	−3.360***	−3.214***
					(0.335)	(0.331)	(0.331)
**Clustering**						0.000816***	0.000846***
						(0.000139)	(0.000139)
**Same neighbors**							−0.000297***
							(4.12e-05)
**Observations**	55,180	55,180	55,180	55,180	55,180	55,180	55,180

Logistic regressions with marginal effects of covariates. The model was performed for the Finnish dataset. Non-survivor firms were excluded. Closeness, betweenness, clustering coefficients, and neighbors in same municipality are in percentages. It must be noted that although closeness and betweenness are in percentage, their empirical range is quite restricted. For example, the firm with highest betweenness has a level of nearly 7%, thus the high marginal effect. Standard errors in parentheses.

*** significant at 1%.

Neighborhood metrics of firms also yield useful information. When firms have higher clustering coefficients, it means that they live in better-connected communities. These firms are more likely to produce positive employment growth. Additionally, if a firm has a higher percentage of neighbors from the same municipality, it is *less likely* to experience positive growth.

Given the evidence that LFNs can be useful to identify employment growth, we proceed to study the contribution of firms to employment growth. We compare employment growth from different groups of firms according to (1) and using standard classifications form the small-business literature and one that considers the LFN (see Materials and Methods). Employment growth in a class of firms is measured as the sum of the net growth of all the companies of that group, in terms of the number of employees. This is a standard procedure in this type of exercise. Using net growth implies that the total employment growth of an economy is measured exclusively through firms that experience positive growth. Therefore, the following results are focused on subgroups of such firms. We found that LFNFs contribute to 88.3% of employment growth in Finland while the second largest contributor –high-growth firms (HGFs)– produce 63.5%. LFNFs represent 7.1% of all firms in Finland. They are more common than HGFs by 0.9%. Employment-wise, LFNFs are more productive (see Table 3).

**Table 3:** Employment by types of firms.

	Labor Flow Network Firms	High Growth Firms	Gazelle Firms	High Impact Firms
**Average firm size**	6.0	1.7	1.7	1.8
**Average firm age**	13.6	11.1	2.7	8.4
**Employment growth share**	88.3%	63.5%	21.1%	32.1%
**Fraction of all firms**	7.1%	6.2%	1.8%	3.1%
**Employment growth per firm**	3.0	2.5	2.9	2.5
**Average incoming labor**	4.3	2.3	3.0	2.4
**Average outgoing labor**	2.2	0.7	0.9	0.7
**Average turnover**	1.8	2.3	2.3	2.3

Firms were classified using the taxonomy presented in the Materials and methods section. Employment growth was measured only for survivor firms using equation (1). Shares are in terms of the total employment growth of the Finnish economy.

**Figure 5:**
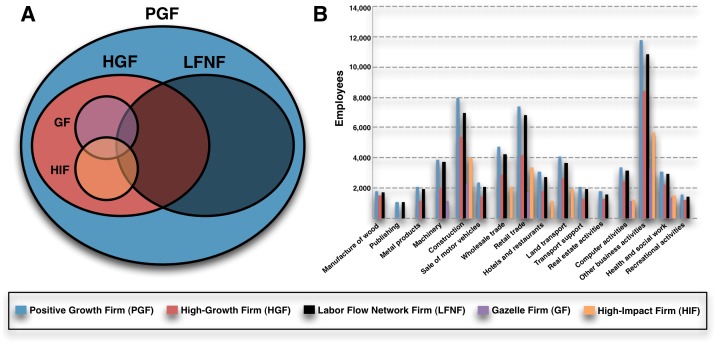
Classification of firms and industrial participation. The Venn diagram is approximately proportional to the number of firms in each category. The bar chart compares total employment of each group in each sector. Industries are classified using the 2-digit European Union's classification of economic activities, NACE.

HGFs, GFs, and HIFs are predominantly composed of firms with initial size zero (about 80%), which explains part of their explosive growth. This proportion drops to 44% for LFNFs. GFs and HIFs are subsets of HGFs. Some LFNFs intersect each one of those groups and some do not belong to any of them (panel A in Figure 5). This means that, on one hand, a subpopulation of HGFs, GFs, and HIFs is heavily composed of isolated companies that do not participate in any flow of workers with any other firm in the economy. On the other, LFNFs might include firms that do not growth so intensively, but that take part in the labor reallocation process. As shown in Figure 5, an average LFNF has six employees and is 13.6 years old, which makes it larger and older than the average firm in one of the other classes. LFNFs receive more workers and also send more labor to other firms. The LFNF turnover rate is of 1.8 workers; 0.5 workers lower than other types of firms. A LFNF produces a net average of three jobs.

From all firms with positive growth (PGFs), only LFNFs are present in all industrial sectors (panel B in Figure 5). They are, consistently, the subclass with most firms and employment across sectors. Therefore, classifications that are based exclusively on growth intensity have the problem of excluding industries that might by important to employment growth through the LFN.

It is evident that firms embedded in the LFN have an important role in employment growth. Considering all the firms in a LFN (not only survivors), they represent 28% of all firms. They are responsible for 90% of employment growth and 91% of its destruction. This implies that the majority of the destroyed jobs are transformed into new ones, most of them filled with people who found their way through the LFN. We next demonstrate that agent-based models are able to generate these dynamics.

### Emergence of Labor Flow Networks

We have shown empirical evidence of complex structures that underlie labor dynamics. In addition to the pure topological properties of the LFN, there are numerous other structures that relate to the characteristics of firms and labor. Many questions regarding causal mechanisms between employment dynamics and LFNs arise from our results. One of special interest to us is how such structures emerge from economic interactions of individuals and firms.

Stochastic processes and game theoretic models are the conventional tools used to explain the formation of networks [46]. Stochastic processes that generate complex networks with multiple stylized facts (degree distribution, clustering, hierarchies, assortativity, etc.) are a few [47], [48]. In the best case, they produce highly stylized statistical properties that do not capture the rich microstructures that we have documented. Furthermore, they contain no economic behavior, which makes them difficult to use for policy purposes. Game theoretic models focus on the behavioral side by providing incentive-driven foundations to the formation of networks [8]–[10]. Usually, these models are *not able* to produce statistical features of complex networks because they simplify the nature of the interactions in order to identify equilibrium outcomes analytically. It is commonly the case that networks are a side product of complex dynamics, and *not* the result of rational decisions for formation of an optimal configuration of connections. Therefore, we believe that the best way to capture the complexity of labor markets is through a bottom-up approach.

**Figure 6:**
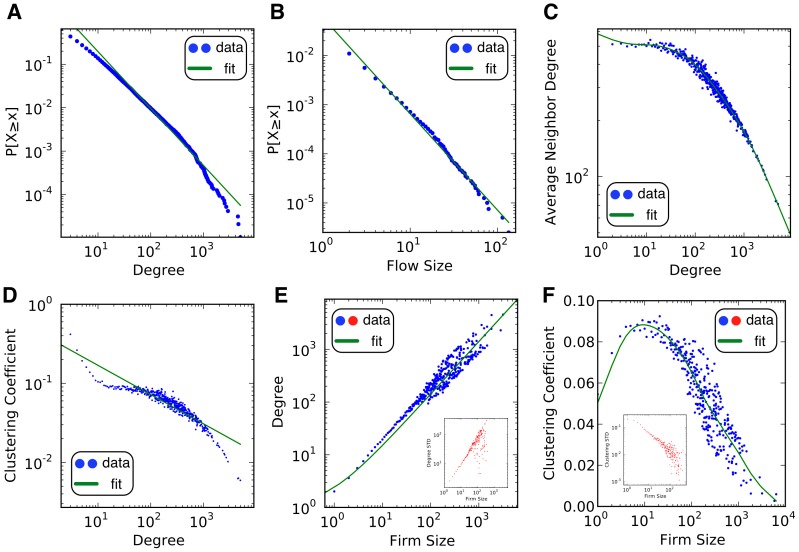
Model output. Data from panels A and B were fitted using maximum likelihood estimation. We used kernel regression to identify critical regions in panel C and F. Fitting in panels D and E were made with OLS. Panels A to D correspond to the results shown in panels A to D in Figures 1 and 2. Panels E and F correspond to the results presented in panels A and C in Figure 3.

We used the agent-based model described in Materials and Methods to simulate the dynamics of the Finnish labor force. The output of the model is presented in Figure 6. Panels A and B match our empirical findings from panels A and B in Figures 1 and 2 regarding the Pareto-distributed degree and labor flow sizes. The model is able to generate the semi-disassortative character of the degree of firms (see panel C in Figure 6 and panel C in Figure 1 for comparison). Surprisingly, it has a transition point near 100 connections where firms with high degree tend to be connected to firms with lower degree. Finally, panel D in Figure 6 shows that the model also gives rise to a hierarchical structure (see panel D from Figures 1 and 2 for a comparison).

An additional challenge of getting LFNs to emerge is generating realistic correlations between economic variables and network characteristics. Stochastic formation processes cannot meet this challenge due to their lack of economic foundations. We found remarkable results regarding the positive correlation between degree and firm size (panel E in Figure 6) with increasing volatility (inner panel), which matches our findings in panel A of Figure 3. Similarly, panel F in Figure 6 matches the non-linear correlation between clustering coefficient and firm size that we documented in panel C of Figure 3. This is an important result since it shows that the model not only generates empirically sound network and economic characteristics at the firm level, but also at the neighborhood level.

This exercise shows the advantage of using agent-based models (ABMs) to generate empirical regularities of labor dynamics. Other type of models might be useful as proof of concept to generate simple relationships between variables. However, we believe that ABMs are a natural way for modeling processes where interactions and distributed micro-dynamics have a central role. This is a first step towards the development of comprehensive models that can be used for policy experimentation. We leave for future research the development of these models and a systematic way to use them for labor policy design.

## Discussion

The network character of labor flows between firms has been investigated and the usefulness of the labor flow network (LFN) concept for the study of firm and employment dynamics has been demonstrated. In many ways such networks have ‘extreme’ properties, in the sense that ‘heavy tails’ characterize many of their empirical features. The dynamics of labor flows in such networks are very far from the ‘smooth’ flows one might expect to occur if any worker could migrate to any company. Clearly, from the structure of empirical LFNs, migration is constrained and the extent to which LFNs deviate from complete graphs is indicative of the magnitude of the ‘lumpy’ and clustered labor flows that can occur in them. Macroeconomic dynamics that are qualitatively similar –also known as ‘granular’– are produced by similar heavy-tailed distributions in firm size [49]. Furthermore, many of the network properties of LFNs have explicit, underlying economic meaning. The regional structure of firm production, along with its demarcation into sectors, are both clearly embedded in LFNs. Perhaps most importantly, signs of employment growth are also present in LFNs and can be determined from network properties. Additionally, many of these properties are common to inter-sectorial networks, that lead to effects on aggregate production fluctuations of production [50]. Finally, using the latest computational advances to model individual agents interactions and the emergence of LFNs, labor policies can be tested artificial laboratory. This new capability suggests that LFNs can be important analytical tools for an improved understanding of the performance and potential of modern economies. Countries would be well-served to collect and make available the kinds of data that facilitate the construction of LFNs.
